# The leaky gut and the gut microbiome in sepsis – targets in research and treatment

**DOI:** 10.1042/CS20220777

**Published:** 2023-04-21

**Authors:** Wiwat Chancharoenthana, Supitcha Kamolratanakul, Marcus J. Schultz, Asada Leelahavanichkul

**Affiliations:** 1 Department of Clinical Tropical Medicine, Faculty of Tropical Medicine, Mahidol University, Bangkok 10400, Thailand; 2 Tropical Immunology and Translational Research Unit (TITRU), Department of Clinical Tropical Medicine, Faculty of Tropical Medicine, Mahidol University, Bangkok 10400, Thailand; 3 Department of Intensive Care and Laboratory of Experimental Intensive Care and Anesthesiology (L.E.I.C.A), Academic Medical Center, University of Amsterdam, Amsterdam, The Netherlands; 4 Centre for Tropical Medicine and Global Health, Nuffield Department of Medicine, Oxford University, Oxford, United Kingdom; 5 Mahidol–Oxford Tropical Medicine Research Unit, Faculty of Tropical Medicine, Mahidol University, Bangkok 10400, Thailand; 6 Department of Microbiology, Faculty of Medicine, Chulalongkorn University, Bangkok 10330, Thailand; 7 Center of Excellence on Translational Research in Inflammation and Immunology (CETRII), Department of Microbiology, Chulalongkorn University, Bangkok 10330, Thailand

**Keywords:** faecal transplantation, gut dysbiosis, leaky gut, lipopolysaccharides, microbiome, sepsis

## Abstract

Both a leaky gut (a barrier defect of the intestinal surface) and gut dysbiosis (a change in the intestinal microbial population) are intrinsic to sepsis. While sepsis itself can cause dysbiosis, dysbiosis can worsen sepsis. The leaky gut syndrome refers to a status with which there is an increased intestinal permeability allowing the translocation of microbial molecules from the gut into the blood circulation. It is not just a symptom of gastrointestinal involvement, but also an underlying cause that develops independently, and its presence could be recognized by the detection, in blood, of lipopolysaccharides and (1→3)-β-D-glucan (major components of gut microbiota). Gut-dysbiosis is the consequence of a reduction in some bacterial species in the gut microbiome, as a consequence of intestinal mucosal immunity defect, caused by intestinal hypoperfusion, immune cell apoptosis, and a variety of enteric neuro-humoral-immunity responses. A reduction in bacteria that produce short-chain fatty acids could change the intestinal barriers, leading to the translocation of pathogen molecules, into the circulation where it causes systemic inflammation. Even gut fungi might be increased in human patients with sepsis, even though this has not been consistently observed in murine models of sepsis, probably because of the longer duration of sepsis and also antibiotic use in patients. The gut virobiome that partly consists of bacteriophages is also detectable in gut contents that might be different between sepsis and normal hosts. These alterations of gut dysbiosis altogether could be an interesting target for sepsis adjuvant therapies, e.g., by faecal transplantation or probiotic therapy. Here, current information on leaky gut and gut dysbiosis along with the potential biomarkers, new treatment strategies, and future research topics are mentioned.

## Introduction

Sepsis is a common syndrome with high mortality and morbidity [1]. Despite recent reductions in sepsis mortality rates, sepsis continues to account for approximately 20% of global deaths, with a staggering 60% mortality rate in patients with septic shock [2,3]. Bacterial infection is the most common cause of sepsis, but most of the clinical manifestations of severe infection caused by bacteria, fungi, viruses and parasitic infection, such as leptospirosis, aspergillosis, dengue shock syndrome and severe malaria, are surprisingly similar, and include cardiovascular dysfunction, resulting in low blood pressure and poor tissue perfusion, renal injury, resulting in anuria, and pulmonary dysfunction, resulting in hypoxemia [4–7]. These similarities imply the possible operation of a predominant innate immune response, i.e., the rapid immune response, rather than adaptive immunity, i.e., the late specific response [8]. The role of microbial molecules not produced by the host (pathogen-associated molecular patterns [PAMPs]) and molecules from the host’s cells (damage-associated molecular patterns [DAMPs]) are distinguished from regular immune homeostasis by innate immunity during sepsis [9]. An adaptive immunity, orchestrated by T and B lymphocytes, along with antibodies, is equally important [10]. Some of PAMPs and DAMPs with the sources and main pattern recognition receptors are listed in Table 1 [11–13].

**Table 1:** Common PAMPs and DAMPs in sepsis

Pathogen-associated molecular patterns (PAMPs)		
Name	Possible sources*	Main PRR**/***
Lipopolysaccharide	Gram-negative bacteria	TLR4#
Lipoteichoic acid	Gram-positive bacteria in gut	TLR2
Flagellin	Filamentous bacteria	TLR5
Lipoarabinomannan	Non-tuberculous mycobacteria	TLR2
Triacyl lipoproteins	Several bacteria	TLR1, TLR2
CpG motifs	Bacteria and viruses	TLR9
β-Glucan	Fungi	Dectin-1, TLR2, TLR4
Zymosan (beta glucan with mannans)	Fungi	TLR2, Several Dectins
Double-stranded RNA (dsRNA)	Viruses	RIG-I, MDA5, PKR (cytosolic receptors) ##
Single-stranded RNA ssRNA	Viruses	TLR7, TLR8

Host damage-associated molecular patterns (DAMPs)		
Name	Possible sources	Main PRR*
RNA	Ruptured host cells	TLR3, TLR7, TLR8, RIG-I
MDA5		
DNA (host and mitochondria)	Ruptured host cells	TLR9, TLR4
Histones	Ruptured host cells	TLR2, TLR4
High mobility group box-1 (HMGB1) and S100 proteins	Secreted from immune cells or cell rupture	TLR2, TLR4, RAGE ###
Heat shock proteins (HSPs)	Host cells with stress	TLR2, TLR4, CD91

*, mostly in gut or respiratory tract; **, PRR, pattern recognition receptors; ***, mostly on myeloid cells; #, TLR, Toll-like receptor; ##, RIG-I, retinoic acid-inducible gene I, MDA5, RIG-I-like receptor dsRNA helicase enzyme, PKR, Protein Kinase R (an interferon-induced kinase); ###, RAGE, receptor for advanced glycation end products.

The importance of PAMPs in sepsis implicates the gastrointestinal tract as an endogenous reservoir of several groups of organisms, including prokaryotes, i.e., bacteria and archaea, eukaryotes, i.e., fungi, and viruses, mostly bacteriophages, which are jointly referred to as ‘gut microbiota’. These organisms are separated from the host by only a single layer of enterocytes containing tight junction molecules [14,15]. During sepsis, enterocytes experience hyperpermeability caused by several factors, including intestinal hypoperfusion, enterocyte apoptosis, a systemic cytokine storm, and gut dysbiosis, which could promote the translocation of microbial molecules from the gut into systemic circulation. This is often referred to as the ‘leaky gut’ [16,17], which is a factor that might be associated with enhanced systemic inflammation in several conditions, either with regular activities (vigorous exercise, high amount of chili, some drugs and stress) [18–20] or pathogenic conditions (autoimmune diseases, infections, obesity and uremia) [21–24]. There are differences in the pathophysiology of leaky gut in these diseases. For example, gut permeability damage in systemic lupus erythematosus (a common autoimmune disease) is possibly due to immune-complex deposition in the gut and the adverse effects of some medications, including nonsteroidal anti-inflammatory drugs (NSAIDs), corticosteroids, and disease-modifying antirheumatic drugs (DMARDs) [14]. Meanwhile, the stress-induced leaky gut is a result of stress hormone-induced immune alteration with autonomic nervous system (gut–brain axis) [25] and both impacts of lupus and stress finally cause gut dysbiosis and leaky gut. The enhanced gut permeability that is sufficiently severe to allow the translocation of viable bacteria, and especially some invasive bacteria, from the gut to the systemic circulation, could be a cause of sepsis, frequently referred to as ‘gut-derived sepsis’ [26,27]. The microbiota, local immunity and integrity in the gut are important factors for the maintenance of the gut microenvironment; therefore, manipulations of these factors might be beneficial in sepsis treatment. Despite increasing knowledge on leaky gut and gut dysbiosis in sepsis, the clinical translation of this information to patients is still very less. Although the alteration of gut bacteria during sepsis is well-known, the exploration of sepsis-induced alteration in fungi and viruses in the gut is recently increasing which might uncover new interesting aspects. Then, the collection of current data on this topic might facilitate interest in the use of some parameters and treatments in real clinical practice. Hence, this review summarizes the potential impact of the gut microbiome, in terms of bacteria, fungi and viruses, on the course of sepsis, and explores currently proposed adjuvant therapies, including faecal transplantation or probiotic therapy.

## A leaky gut leads to the presence of microbial molecules in the systemic circulation

A single layer of epithelial cells with a surface area of about 32 m^2^ lines the gastrointestinal (GI) surface and is held together by epithelial tight junctions (TJs). This layer functions as the first stage of the intrinsic mucosal defence system and serves as a selective physical barrier between the host and microbial molecules [28,29]. The TJ complex does not allow the passage of molecules larger than 3.6 Å or 0.6 kDa through the normal paracellular passage (the space between the proximity of enterocytes). The larger molecules are transported through the gut epithelial cells by several transcytosis mechanisms, including clathrin-mediated endocytosis, micropinocytosis and caveolin-mediated endocytosis [30,31]. Some microbial-derived molecules, such as p-cresol (a gut-derived uremic toxin derived from protein fermentation by gut bacteria), are small enough to pass through the normal gut barrier [32], whereas other molecules, such as lipopolysaccharide (LPS) from Gram-negative bacteria and (1→3)-β-D-glucan (BG) from fungi (the most abundant and second most abundant organisms in the gut) or microbial DNA, are too large to cross the barrier [33]. However, although large intact bacterial DNAs (i.e., the genome) with molecular sizes of 100 to 15,000 kilobase pairs (kbp) (6.5 × 10^4^–9.8 × 10^6^ kDa) are too large to pass through the gut barrier, DNA molecules are rapidly broken down into bacteria-free DNA through several processes (depurination and deamination) into pieces approximately 100 bp (65 kDa) in size (i.e. similar in size to LPS and BG) [34]. Hence, the detection of these PAMPs (LPS, BG and bacteria-free DNA) could be useful indirect markers of leaky gut. Alternatively, the oral administration of a non-absorbable carbohydrate and its subsequent detection in blood or urine is a well-known direct test for leaky gut [35,36]. However, the necessity of oral administration and intact intestinal peristalsis limits the use of this procedure only to patients in non-moribund conditions.

Local intestinal injury with a large surface area does not surprisingly induce leaky gut, as demonstrated in mice treated with a low concentration of dextran sulphate solution (DSS), a substance that directly causes TJ injury. The intestinal symptoms of leaky gut progress from asymptomatic to overt diarrhoea [37] or acute pancreatitis with endotoxemia [38]. In parallel, high-abundance PAMPs detected in serum, including in DSS-administered mice, are indicators of leaky gut [39]. Interestingly, leaky gut in DSS-administered mice is demonstrable with a fluorescein isothiocyanate (FITC)-dextran assay. In humans, the detection of some non-absorbable carbohydrates in urine after an oral administration is demonstrated, even without abdominal symptoms (diarrhoea or abnormal stool consistency) [40], implies a possible asymptomatic leaky gut. As such, the current hypothesis is that ‘a physiologic leaky gut (leaky gut without a significant adverse effect)’ may exist, as is observed in blood microbiome analyses with the presence of DNA from anaerobic gut bacteria that are usually not present in the blood circulation [34]. Although the abundance of DNA in the blood of healthy control mice is very low or non-detectable, the DNA amplification processes used in bacteriome analysis can detect low amounts of DNA. Notably, the regular repairing process of the ‘physiologic leaky gut’ should not produce intestinal fibrosis due to the prominent self-renewal property of the enterocytes [41]. However, intestinal fibrosis can be developed in case of severe overt chronic inflammation as reported in inflammatory bowel disease (ulcerative colitis) [42].

Further possible evidence for a physiologic leaky gut is the detection of serum BG in some healthy people, especially with the Fungitell assay (Associates of Cape Cod, Inc.), as BG is a major component of fungi that is foreign molecule to the host, with a normal range (less than 60 pg/ml) that possibly reflects a leaky gut in healthy individuals (detectable serum BG without detrimental condition) [17,35,36]. As such, BG is the natural polysaccharide consisting of sequential D-glucose moieties linked by β-(1→3)-glycosidic bonds with other structural varieties depending on the sources, such as BG from fungi is composed of β-(1→6)-linked branches from the β-(1→3) backbone [43]. Proinflammatory impacts of BG, especially in synergy with LPS, are frequently mentioned [44–48].

By contrast, endotoxaemia should not be detectable in a healthy host, despite a possible low level of leaky gut, perhaps due to several LPS neutralization actions, such as deacylation and dephosphorylation by acyl-oxy-acyl hydrolase and alkaline phosphatase, respectively [49–51]. Notably, no enzymatic reaction exists for BG neutralization [52]. Hence, LPS and BG in serum, in the absence of other obvious sources, are interesting leaky gut biomarkers that are more practical for clinical use compared with the standard oral carbohydrate administration. However, the level of LPS and BG in serum not only depends on the severity of a leaky gut, but also correlates with increased numbers of Gram-negative bacteria and fungi in the gut. In animal models, several conditions lead to an increase in Gram-negative bacteria (Bacteroides and Proteobacteria) and possibly LPS in gut contents, including sepsis, DSS-induced mucositis, uraemia, obesity and fungal administration [39,48,53–55], while an enhanced content of faecal fungi (and BG) in the gut is possible after antibiotic use, intestinal inflammation (inflammatory bowel disease; IBD), and alcohol consumption [44,45,56–58]. Thus, using the actual quantity levels of LPS and BG to determine the severity of leaky gut is difficult; however, they might be useful for qualitatively indicating gut barrier damage.

Observing leaky gut is not surprising after acute or chronic diarrhoea from any causes (infection, immune-mediated diseases, and DSS) [53,59–61] due to direct damage to the TJ. However, the pathophysiology of systemic inflammation-induced leaky gut might involve inflammation-induced paracellular enterocyte permeability (as demonstrated by LPS injection models) [62] and/ or stress-induced gut dysbiosis [63,64]. Indeed, LPS injection triggers the production of serum cytokines, which can affect every cell in the body, including enterocytes, and cytokine activation worsens enterocyte integrity, as demonstrated by the reduced transepithelial electrical resistance in enterocytes after incubation with pro-inflammatory cytokines [65]. In addition, neuro-hormonal disturbances in response to stress (and depression), especially the enhancement of catecholamine, can alter the bacterial composition in the gut, in part, due to catecholamine iron chelation that facilitates the growth of iron-metabolizing bacteria [66]. The activation of enteric neurons by corticotropin-releasing factors in immune cells (macrophages and mast cells) can also alter the microbial control mechanism in the gut [67]. It is also interesting to note that there is a balance of the immune responses, referred to as the ‘counter anti-inflammatory response’, during the hyper-inflammatory activity in severe systemic inflammation, especially in sepsis, the imbalance homeostasis of immune regulation seems to induce either hyper-inflammatory septic shock or immune exhaustion (an increased susceptibility to secondary infection) [68–70] that might be able to cause enterocyte injury and leaky gut, perhaps, with different processes. More studies on this topic would be interesting.

## Leaky gut and gut dysbiosis

The balance between the host immune activities and micro-organisms in the gut leads to the specific characteristic of gut microbiota in different hosts as genetic-based immune responses and gut microenvironmental aspects (diets and regular activities) might be different among individuals. As such, an alteration of immune activities in the host, due to aging, antibiotics, foods, or the new onset of some systemic diseases possibly results in a change in gut microbiota [71–73]. For example, the depletion of macrophages or splenectomy in the host reduces the microbicidal activity against some gut organisms leading to gut dysbiosis [64,74] and the selective microbicidal activities of different antibiotics induce some different dysbiosis in the host [75,76]. In contrast, gut dysbiosis might induce some changes in immune responses that possibly affect intestinal integrity. As such, gut dysbiosis induced by oral administration of pathogenic bacteria or fungi facilitates a direct invasion of enterocytes and activates the more prominent immune responses leading to a more severe leaky gut than the presence in the host with lesser harmful microbes [37,45,77]. Notably, the presence of gut fungi alters the composition of gut bacteria through several mechanisms, such as a selection of bacteria that can digest some molecules on fungal cell walls or bacteria with fungal toxin resistance [39,74]. Hence, immune activities, both local intestinal immunity and systemic immune responses, affect gut dysbiosis and vice versa that can cause defects in the intestinal barrier (leaky gut) through the damage by immune responses (enterocytes are the bystanders from microbicidal immunity) and/ or from the invasiveness of the pathogenic microbes.

During sepsis, there was an alteration in immune responses and gut dysbiosis with several sepsis factors that enhances intestinal barrier defect. For sepsis-induced immune responses, hyper-inflammatory cytokines, death of immune cells from the overwhelming immune activities and stress hormone-activated intestinal immunity [8,78,79] that might affect the normal balance between the host immunity and microbes. In sepsis-induced gut dysbiosis, an abundance of the high virulence organisms in the gut during sepsis might be increased because these bacteria usually have several factors against the harsh microenvironment, while normal microbiota mostly demonstrated a lack of these factors [80]. Moreover, several defects during sepsis, for example, gut hypoperfusion from systemic vasodilatation and/or sepsis-induced cardiomyopathy, intestinal hypomotility and gut mucosal disruption [81] also directly induce gut barrier defect and leaky gut. Hence, enterocyte hyperpermeability in sepsis is caused by several factors, including intestinal hypoperfusion, enterocyte apoptosis, systemic cytokine storm and gut dysbiosis that could promote the translocation of microbial molecules from the gut into the blood circulation (leaky gut or gut leakage) [16,17].

Although the endotoxemia and circulating cell-free DNAs (cf-DNAs) observed in bacterial sepsis might be derived from dead bacteria in the blood, some LPS molecules might be correlated with translocation from the gut into the blood circulation (gut translocation). Better evidence of leaky gut during sepsis comes from the presence of endotoxemia and glucanemia (serum BG) without bacteraemia during viral sepsis, such as is observed with dengue and coronavirus disease 2019 (COVID-19) with high disease severity [35,82–84]. Although a mixed bacterial–viral infection is possible, antibiotics (and anti-fungal) are not necessary for most of these patients with severe viral sepsis. Additionally, the administration of bacterial lysate also containing bacterial DNA during leaky gut induction by DSS in mice increases the level of cf-DNAs in the blood [34], implying a possible gut translocation during sepsis. One interesting finding is that leaky gut could be a cause and/or consequence of bacterial sepsis because (i) the severe gut barrier defect induces viable bacterial translocation and bacteraemia, as indicated by DSS-induced sepsis [64,77], and (ii) the damage to the enterocyte TJ during sepsis facilitates leaky gut [45]. In both situations, the leaky gut enhances systemic inflammation through innate immunity responses, especially through macrophages and neutrophils [40,46,85]. Similarly, gut dysbiosis (the imbalance of gut microbiota associated with an unhealthy outcome) can be a cause and/ or a consequence of bacterial sepsis due to the importance of gut microbiota in the maintenance of intestinal integrity [86].

Currently, several methods (multi-sugar probe, LPS, BG and other molecules) [87] are available for leaky gut measurements, but performing these measurements during sepsis is challenging because of the limitations in oral carbohydrate administration to critically ill patients, the possible differences in the abundance of LPS and BG in the gut contents, Gram-negative bacteraemia (which limits the use of LPS as a leaky gut marker) and the unclear clinical usefulness of several molecules (zonulin, fatty acid binding protein and others). Due to high susceptibility to the leaky gut during sepsis, a quantitative test of leaky gut might not be necessary, and qualitative tests for leaky gut (such as BG) with dysbiosis indicators (such as the abundance of Firmicutes, Bacteroides and Proteobacteria by microbiome analysis or polymerase chain reaction [PCR]) might be adequate for clinical use. Although species differences detected by microbiome analysis are more informative, the differences in phylum levels using PCR with selected primers might be less expensive and more suitable for real clinical use. More studies on this topic are warranted.

## The intestinal bacterial microbiome

Because bacteria are the most predominant organisms in the gut, most of the ‘gut dysbiosis’ mentioned in the studies predominantly refers to bacterial dysbiosis. The normal gut microbiota includes a predominance of Firmicutes (Bacillota) (mostly Gram-positive bacteria with obligate aerobes or facultative anaerobes) and Bacteroides (mostly Gram-negative anaerobes that are pathogens in some situations) [88]. Firmicutes are the most prominent bacteria in the healthy gut, in part due to the conversion of complex carbohydrates into short-chain fatty acids (SCFAs, particularly butyrate), which are important growth factors for the gut epithelium. Bacteroides are the most dominant Gram-negative bacteria in the gut and possibly represent a major source of LPS in the intestine [89]. The ratio of Firmicutes/ Bacteroides could serve as a biomarker for the health of the gut barrier, as this is lower in several conditions, including infection, DSS colitis, post-splenectomy, macrophage depletion, obesity, uraemia, iron overload and sepsis [24,48,55,77,90,91], and an increased Firmicutes/Bacteroides ratio is reported in IBD [92,93]. Despite the benefits of SCFA production by most Firmicutes bacteria (such as the probiotic strains of lactobacilli and enterococci), some groups (such as a subset of clostridial species) are pathogens that might induce gut barrier damage [94,95]. Likewise, several species of Bacteroides bacteria supply nutrients to other microbial residents and reduce pathogens in the gut, despite the possible pathogenicity of other Bacteroides [88]. Proteobacteria (Pseudomonadota), a major phylum of Gram-negative bacteria (including a wide variety of pathogens), is another bacterial phylum that frequently shows increases during gut dysbiosis [96–98]. Thus, both increases and decreases in the Firmicutes/Bacteroides ratio with increased Proteobacteria indicate gut dysbiosis; however, more studies are warranted before adopting this ratio for clinical use.

The normal gut microbiota is vulnerable to the microenvironment, as the oral administration of bacteria or fungi causes leaky gut from an increase in pathobionts [37,45], while leaky gut due to DSS induces dysbiosis through gut mucosal inflammation [53]. Intestinal inflammation might therefore be another factor that induces gut dysbiosis, as the oral administration of *Candida albicans* in control mice does not alter faecal microbiota patterns, while *C. albicans* gavage in septic mice after cecal ligation and puncture (CLP) surgery or DSS-colitis increased the proportion of Gammaproteobacteria (a group of pathogenic bacteria, including *Pseudomonas aeruginosa*) [39,53]. Indeed, intestinal inflammation from several causes, including some diets (high-fat diets), drugs (non-steroidal anti-inflammatory drugs; NSAIDs) and stresses (heavy exercise), can reduce mucin production (mucin barrier) and increase the number of pro-inflammatory cells (and mediators), resulting in a selection of some groups of bacteria that are more resistant to host immunity (mostly the highly virulent pathogenic bacteria) [18,19,99–101]. Conversely, the reduction in immune responses, such as macrophage depletion, also possibly increases some bacteria that are naturally controlled by intestinal macrophages and causes gut dysbiosis [74].

Due to the vulnerability of gut microbiota, some host characteristics might be theoretically classified as sepsis-vulnerable features. This could occur in individuals with a lower abundance of SCFA-producing bacteria, a genetic deficiency in the normal gut barrier (production of mucin and anti-microbial peptides; AMPs), or in those with malnutrition or immunodeficiency, as the faecal microbiome is a sensitive biomarker for these conditions [102,103]. For example, Mucin 2 deficient (Muc2-/-) mice develop colitis at 6 months of age, with increases in Firmicutes/Bacteroidetes and some Proteobacteria (*Desulfovibrio* and *Escherichia*) [104]. A defect in AMPs is mentioned in IBD-induced dysbiosis [105], and children with severe acute malnourishment demonstrate increased Proteobacteria and decreased Bacteroides in faeces [106,107]. Therefore, a reduction in Firmicutes or a low Firmicutes/Bacteroides ratio might be an indicator of low numbers of SCFA-producing bacteria and might represent a characteristic of susceptibility to gut-derived sepsis because of the easier gut invasion of pathogenic bacteria [93,108]. However, detection of the possible adverse bacterial groups in healthy individuals might not be clinically significant because of the other intact protective factors (such as mucin and intestinal immunity). Moreover, the organismal molecules from a transient leaky gut, even a severe one, might be quickly neutralized by several processes similar to those occurring in the physiological leaky gut. Therefore, measurements of the leaky gut at several time points might be necessary to identify a representative and clinically significant leaky gut in real patients, as this might differ from animal models that have less fluctuation in conditions.

Our experiments have indicated that spontaneous bacteraemia in some acute uremic mice after 48 h bilateral nephrectomy is possibly caused by intestinal apoptosis, which leads to severe leaky gut [90], again implying the importance of the gut barrier. Although the prediction of sepsis susceptibility by gut dysbiosis alone, or perhaps by the reduction in Firmicutes (or increases in Bacteroides and Proteobacteria) without leaky gut measurement, might provide limited information, several reports support some predictive properties of dysbiosis. For example, the depletion of *Rosburia* (phylum Firmicutes) and increases in *Prevotella* (Phylum Bacteroides) in the gut are identified risk factors for stroke-associated pneumonia and chronic obstructive pulmonary disease (COPD), respectively [109,110], while increases in *Klebsiella variicola* and Enterobacteriaceae (phylum Proteobacteria) are associated with sepsis cardiomyopathy [111]. Notably, some bacterial metabolites, mostly derived from the digestion of nutrients (such as polyamines), are small enough to pass through the normal gut barrier; however, the impact of these molecules in sepsis is not as clear as that of the larger microbial molecules (LPS, BG and cf-DNA) [112,113].

In contrast to the intact gut barrier in dysbiosis before sepsis, sepsis leads directly to gut dysbiosis together with the leaky gut and allows the translocation of microbial molecules or viable microorganisms. The viable microbial translocation from the gut are mostly bacteria rather than fungi (*Candida* spp.), due to the larger size of fungi than bacteria. Reduced intestinal perfusion can be recognized in the early phase of sepsis with normal blood pressure (pre-shock stage), despite systemic vasodilatation (distributive shock) and myocardial depression (partly from hyper-cytokinaemia) [114,115], by a decrease in gut microcirculation as presented by sepsis-induced ileus [116]. Because ileus can be an early sign of systemic inflammation, either from infection (sepsis) or non-infection (multiple injury or multi-organ failure; MOF), but presents with normal blood pressure, the reduced gut perfusion in sepsis and MOF might occur very early in the natural course of diseases [117]. Among several factors associated with sepsis-induced intestinal disorders [81], gut hypoperfusion is an important factor that possibly results in (i) enterocyte damage (necrosis and apoptosis) with leaky gut and (ii) intestinal immunity defects (the death of immune cells) with decreased microbial control function and increased gut dysbiosis (the selection of only highly virulent bacteria). Sepsis is accompanied by apoptosis of all immune cells (neutrophils, macrophages, dendritic cells and lymphocytes), in part, due to the overwhelming immune activation by both PAMPs from the organisms and damage-associated molecular patterns (DAMPs) arising from the death of the host’s cells [118]. This immune cell apoptosis is one of the mechanisms that induce immune exhaustion (a reduced ability to prevent other infections, leading to secondary infections) [119]. Sepsis also causes dysfunctions in multiple organs (kidney, liver, lung, spleen and nervous system) and the damage to each organ can further affect gut dysbiosis. For example, kidney and liver damage during sepsis might lead to the excretion of accumulated metabolites (toxins) into the gut, and these could directly affect enterocytes and stimulate the growth of some bacteria (such as bacteria that can metabolize these toxins), resulting in dysbiosis with leaky gut [90,120]. Likewise, sepsis can possibly alter immune responses, such as elicitation of lung-produced type I interferons, that can directly alter the gut microbiome [121], thereby possibly reducing the numbers of obligate anaerobic bacteria and increasing the proportion of Proteobacteria [122]. Similarly, alteration of the neuro-immuno-endocrine axis during sepsis might also affect gut dysbiosis [113]. Hence, sepsis induces gut dysbiosis through effects on gut hypoperfusion, immune dysregulation and organ failure.

**Figure 1:**
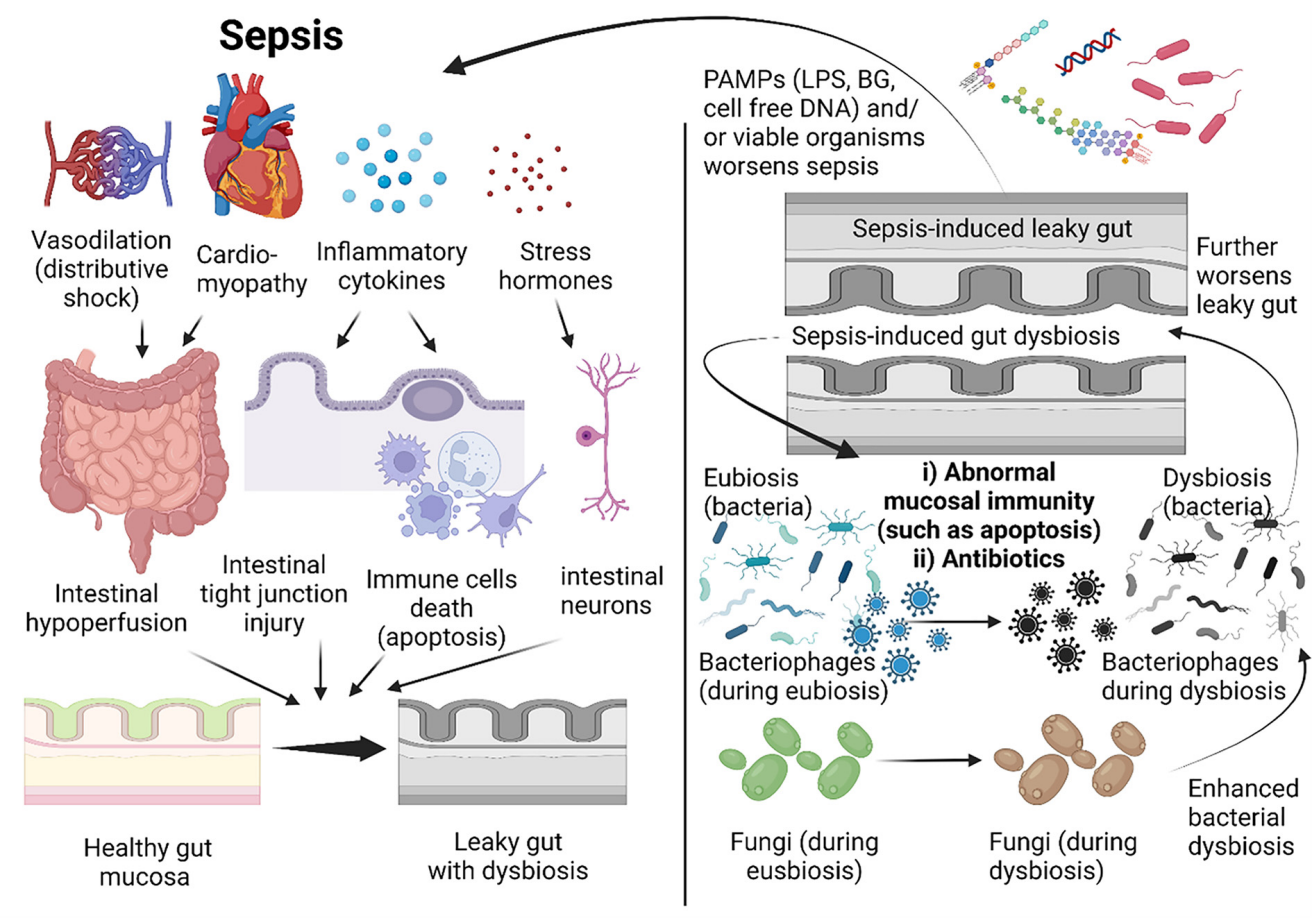
The alteration of all organisms (bacteria, fungi and phages) involved in sepsis and gut immunity Sepsis induces intestinal immunity defects, through intestinal hypoperfusion (vasodilatation and cardiomyopathy), immune cell apoptosis, the stress hormone (corticotropin)/enteric neuron-induced immune responses, and systemic inflammation, inducing gut dysbiosis (left side). In parallel, sepsis-induced gut dysbiosis, caused by intestinal immunity defect, antibiotics and alteration in fungi and phages, facilitates gut translocation of microbial molecules or viable organisms (leaky gut) causing systemic inflammation (right side) that worsen gut integrity and induce gut dysbiosis as a vicious cycle. Picture is created by BioRender.com.

Interestingly, some similarities are evident between sepsis that arises from several different sources of infection. This is due, in part, to common factors among the critically ill and in systemic inflammatory response conditions, including the loss of possible beneficial bacteria and microbial diversity and an increase in pathogens [123,124]. For example, faecal microbiota in children with sepsis contain higher proportions of pathogens (*Acinetobacter* and *Enterococcus*) with fewer beneficial bacteria (*Roseburia*, Bacteroides, *Clostridia, Faecalibacterium* and *Blautia*), and these changes closely correlate with the clinical characteristics but show negative associations with the duration of antibiotics [125]. Similarly, depletion of Lachnospiraceae, Ruminococcaceae and *Ruminococcus* and an enhancement of *Enterococcus* are demonstrated in a systemic review of sepsis [126]. Severe viral infections (COVID-19, influenza and dengue) can also increase pathogen numbers, especially Gram-negative bacteria, during sepsis and facilitate gut translocation of LPS (endotoxaemia) or viable bacteria (bacteraemia), depending on the leaky gut severity, that worsen the severity of infection [127–129] (Figure 1).

## The intestinal mycobiome

Despite the larger size of fungi (10–12 µm; *Candida* yeast) than bacteria (0.5–2 µm), fungi are the second most abundant organisms in the gut. As such, the abundance (by gene copies) is 1000-fold greater for bacteria (16S rRNA) than fungi (18S rRNA), with more than 3,500 bacterial species compared with 267 fungal species in the gut [29]. The bacterial community varies in quantity and composition from the stomach to the colon (10^2^ vs 10^11^ cells/ gram content in the stomach and colon, respectively), whereas fungi seem to be localized mostly in the colon, with an average of 10^6^ fungal cells per gram of colon content [130]. The predominant intestinal fungal mycobiota in healthy individuals are from the phyla Ascomycota (63%) (especially *Candida albicans*) and Basidiomycota (32%) [131], and the overgrowth of *C. albicans* commonly found in patients with bacterial sepsis arises in part due to antibiotic selective pressure [132]. *Candida* colonization in the gut is also an important risk factor for systemic candidiasis after bacterial sepsis [133]. Indeed, *Candida* colonization in the gut is very common in patients in intensive care units (ICUs+) [134,135], and *Candida* translocation from the gut into blood circulation is possible during bacterial sepsis [136,137]. Due to the lower abundance of fungi in mouse faeces than in human stool (positive culture is easier found from humans), the administration of *C. albicans* to mice is used to explore the importance of *Candida* in sepsis. Despite its lower abundance, the presence of *Candida* in the gut enhances some bacterial species (such as *Pseudomonas* spp.) [44,53], partly due to glucan digestion, as mixing glucan into the culture medium enhances the growth of isolated bacteria [39]. Interestingly, the fungal–bacterial interaction is complex and might depend on the time frame of the exposure, as the incubation of a clinical strain of *Pseudomonas aeruginosa* with *C. albicans* has no synergy on biofilm production, whereas the addition of the fungi onto *Pseudomonas* biofilms or onto cell lines facilitates more biofilm production [138,139]. Nevertheless, increases in *Candida* in the gut during sepsis possibly worsens the severity of bacterial sepsis through several pathways, including a higher translocation of BG (*Candida* increases the BG gut content), increases in invasive bacteria in the gut and direct injury to enterocytes (perhaps from the *Candida* germ tube or mucosal immune responses against fungi) [44,77]. Notably, the co-presentation of LPS and BG synergistically activates macrophage immune responses, in part through the simultaneous activation of TLR-4 and dectin-1 by LPS and BG, respectively [45,46,85].

Despite the lack of information regarding the gut mycobiota in patients with sepsis, septic mice demonstrate subtle changes in gut fungi (the abundance of fungal 18sRNA by PCR in sepsis is different from the control group), including a reduction of only *Myrothecium* spp. fungi that can produce some molecules against several harmful factors (some organisms and toxic substances) [15]. The differences in sepsis conditions between humans and mice [140,141] raise the possibility that gut fungi in patients with sepsis might be enhanced by several factors that differ from those in mice, such as the duration of sepsis (human patients survive longer than mice), antibiotic use (more potent in human conditions), intensive care unit (ICU) environment (nosocomial infections are likelier in patients in ICUs than in mice in controlled animal facilities) and naturally higher *Candida* levels in human faeces and underlying diseases (such as altered gut fungi in Type 2 diabetes) [142–144]. Based on the well-established increase in gut fungi in patients with IBD and alcohol ingestion [56–58], intestinal inflammation and reduced mucosal immunity might be important exacerbating factors for the enhancement of gut fungi associated with sepsis (systemic cytokine-induced intestinal barrier defects and apoptosis of immune cells) [65,118]. More exploration of gut fungi in patients with bacterial sepsis will be interesting. Of note, the identification of mycobiota at the phylum level might provide only limited information because Ascomycota predominate; therefore, faecal microbiome analysis might be necessary to explore fungal population in faeces.

## The intestinal virobiome

Currently, viruses in the gut are not included as ‘gut microbiota’, as viruses are intracellular organisms and the presence of viruses in enterocytes will be categorized as a viral infection. However, bacteriophages, which are viruses (or genomes) of the gut bacteria, might be considered a group of viruses that can be found in the gut content and categorized as ‘gut microbiota’ because alteration in gut bacteria will automatically change the abundance of bacteriophages (or phages). Phages are specific to the species level of bacteria, partly because of different routes of entry, and phages of the same bacteria might have different responses to different bacterial isolates [145]. For example, the effective phages against *P. aeruginosa* from Person A might have no effect against *P. aeruginosa* from Person B. This will necessitate a tremendous accumulation of phage information (phage library) for any real clinical use [146].

The bacteriophage cycle is categorized into lysogenic and lytic patterns. The lysogenic cycle involves the insertion of viral genetic materials into the bacterial genome for replication together with the bacteria. These phages are referred to as ‘temperate phages or prophages’ and can be transferred to several bacterial generations without any viral gene expression. By contrast, the lytic cycle is a switch from the lysogenic phase to the release of new viral particles [147,148]. Because phages are one of the natural controls against bacteria [149] and because prophages can pass through several generations of bacteria before being induced (e.g. by stress) into lytic phages and killing the bacteria [150], any alteration in the bacterial microbiome during sepsis might automatically induce changes in virota (virome). Indeed, the faecal virota from septic mice demonstrates an alteration in the abundance of several groups of bacteriophages, including Myoviridae (in sham mice) and Podoviridae (in septic mice), which are components of several phage cocktails used in other studies [15]. The observation that viral particles isolated from faeces of a septic mouse can attenuate sepsis in another mouse [15] raises the possibility that bacterial stress during sepsis activates lytic phages that might be able to control some sepsis-induced pathogenic bacteria. Phages accumulating within the mucosal layer can be a barrier to bacterial invasion; however, bacteria that express phage-encoded proteins can show increased virulence (epithelial invasion, adhesion, antibiotic resistance, phagocytosis blockage and biofilm formation) and the transport of phages by transcytosis of phage particles and/or apical-basal transport may deliver phages into the circulation and enhance inflammatory responses [151,152]. Unfortunately, studies on gut virota (or phageomes), especially in sepsis, are scarce.

## Adjunctive therapies

**Figure 2:**
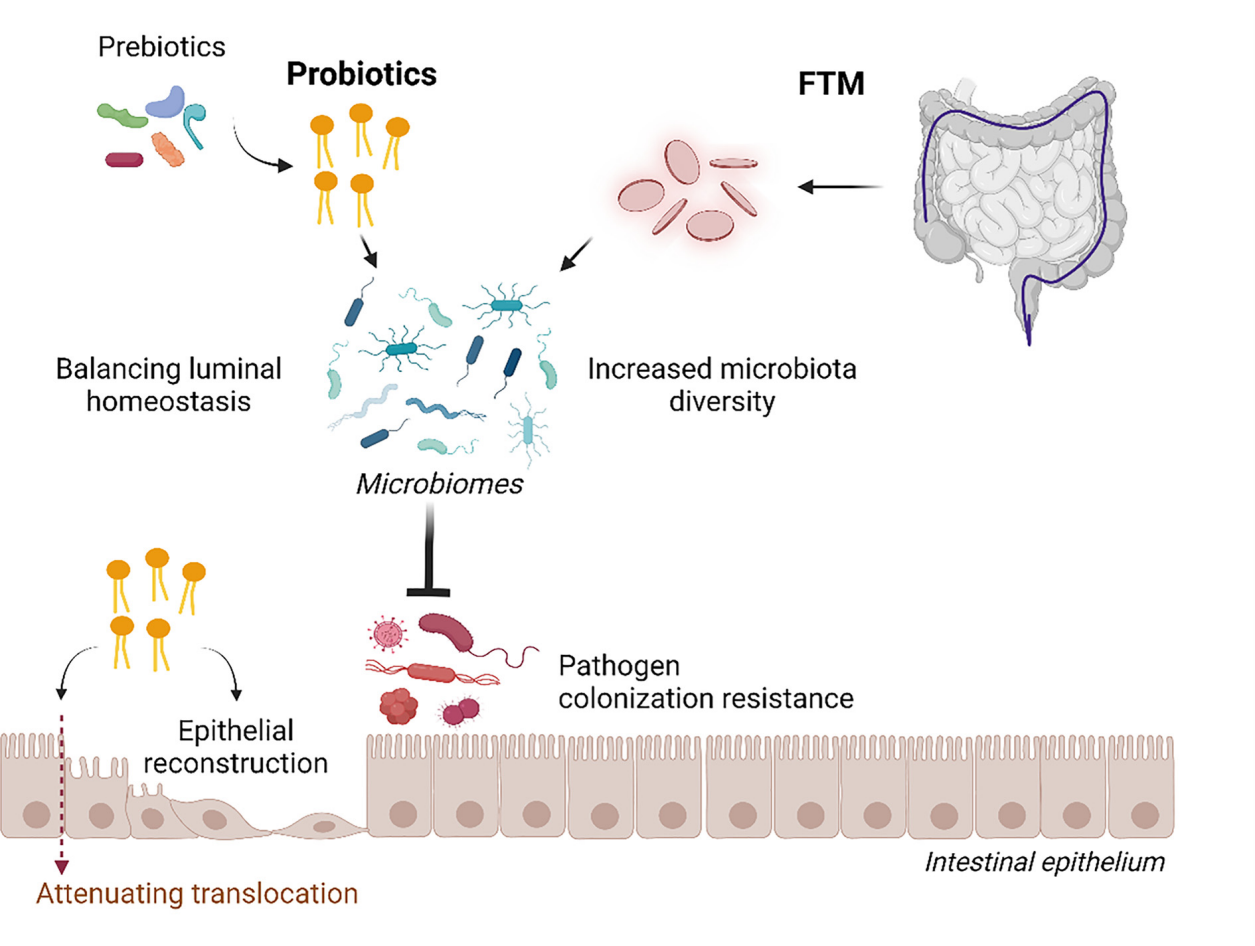
The adjunctive therapy of prebiotics, probiotics and FMT in terms of intestinal permeability effects All of these strategies improve the balance of gut microbiota with increased organismal diversity that is beneficial to the host through reduced pathogenic microbes, strengthens the gut barrier and induces gut epithelial reconstruction.Picture is created by BioRender.com

Due to the possible correlation between gut dysbiosis and sepsis severity, the manipulation of the gut microbiome (and gut barriers) might prevent gut-origin sepsis or attenuate sepsis severity by strengthening the gut barrier, reducing gut pathogens, reducing the PAMP content (LPS and BG) in the gut and eliciting direct anti-inflammatory responses. The normalization of gut microbiota by several methods, including faecal transplantation (administration of healthy microbiota), probiotics (beneficial bacteria) (Figure 2), prebiotics (probiotic-enhancing substances) and synbiotics (probiotics with prebiotics), has been tested in sepsis.

### Faecal microbiota transplantation

Several animal studies and case series have reported the ability of faecal microbiota transplantation (FMT) to attenuate sepsis severity, in part through the restoration of butyrate-producing bacteria, gut barrier strengthening, innate immunity enhancement, immune repertoire alteration and pathogen clearance; however, some studies have reported lethal bacteraemia [153]. Notably, the immune repertoire is a variety of receptors on T cells and B cells that has a large sequence diversity to recognize different organismal molecules as a part of the adaptive immune system [154] and innate immunity, for example, macrophages, is an important host response against pathogenic gut organisms [74]. Meanwhile, butyrate is an important short-chain fatty acid that is categorized as an enterocyte energy source and a factor of anti-inflammation and anti-malignancy [155]. Then, FMT administration seems to enhance the effectiveness of organismal control in the host through improved innate and adaptive immunity together with strengthened enterocyte integrity that will be beneficial in sepsis [156]. *Clostridium difficile* seems to be the first pathogen with FMT clinical implications. *C. difficile* is classified as a Gram-positive bacterial pathogenic cause of infectious colitis that frequently arises following excessive antibiotic use [157]. *C. difficile* contributes to complications of antibiotic therapy owing to its recurrent infections. Interestingly, the use of FMT by either oral pills or FMT colonoscopy in patients with recurrent *C. difficile* showed promising outcomes (96.2% and 96.1% of patients were cured after 12-week treatments by oral FMT and colonoscopy FMT, respectively) [158]. The more updated implications of FMT now involve its applications as cancer therapeutic. This potential as a therapy was first observed in mice with cancer but no microbiome, as these animals demonstrated a different response when treated with anticancer drugs, including cisplatin, cyclophosphamide, and anti-programmed cell death 1 protein (PD-1) immunotherapy [159,160]. These findings are also supported by evidence that *Enterococcus faecalis* is able to directly metabolize levodopa [161]. As such, using gut microbiota in conjunction with drugs could benefit the balance of gut microbes, thereby simultaneously suppressing gut pathogens during certain disease treatments. Nevertheless, in mid-2019, the U.S. Food and Drug Administration (FDA) announced that FMT therapy should be used with serious caution, based on a mortality case report of extended-spectrum β-lactamase (ESBL)-producing *Escherichia coli* infection [162]. As a result, the FDA has posted a warning statement that in-depth screening for all resistant pathogens must be performed before FMT.

### Probiotics

In contrast to the possibly severe side effects of FMT in sepsis treatment, the adverse effects of probiotics are usually minimal, as most probiotics are anaerobes and anaerobic bacteraemia is not normally severe and is easier to treat compared with aerobic bacteriaemia [163]. Probiotics consist of PAMPs; thus, gut translocation of probiotics or their components can activate innate immune responses. Therefore, the administration of probiotics to immunocompromised individuals or those of extreme age, critically ill or with severe leaky gut could cause bacteraemia [164,165]. In some conditions, with appropriate probiotics, the leaky gut might be advantageous because some relatively large beneficial molecules from probiotics might possibly be transported through the damaged gut barrier [54,55]. Probiotics potentiate colonization resistance through thier functions of reduced luminal pH, antimicrobial properties, and competing for nutrients and adhesion surface [36,37]. Indeed, some strains of *Lactobacillus* and *Bifidobacterium* produce some exopolysaccharides with immunomodulatory effects [166,167], while also reducing pathogens by nutrient competition, quorum sensing antagonists and production of substances that directly inhibit bacteria [168]. Several bacterial strains are choices for probiotics, but some bacteria might be more harmful than others. For example, enterococci can cause endocarditis in some conditions, while lactobacilli and *Bifidobacterium* are easily treatable [169]. Probiotics also enhance gut barrier function through mucin production and tight junction proteins. Now, probiotics are extended to other uses, including skin protection from various host pathogens, such as *Staphylococcus*, *Corynebacterium* and *Propionibacterium*, but this use can lead to the development of skin immune disorientation conditions, such as rosacea [170]. Interestingly, local application of probiotics improved skin colonization by *Cutibacterium acnes* [171]. In addition, oral forms of probiotics, such as *Lactobacillus reuteri*, demonstrated an ability to attenuate perifollicular inflammation by promoting a gut–brain–skin (GBS) axis [172].

### Prebiotics

The rationale for the use of prebiotics in leaky gut syndrome is certain dietary components might promote the growth of certain gut bacteria strains that are closely associated with health benefits for the host [173]. Prebiotics are not only the food components non-digestible by the host that promote the fermenting bacteria in the colon [174] but also are nutrients degraded by the gastrointestinal microbiota that alter the microbiome's activity and composition [175]. Many kinds of dietary nutrients are termed prebiotics under these categorizations, especially the commercially available carbohydrate-based dietary fibers (polymers of monosaccharides), which are fermented by intestinal microorganisms. These nutrients are digested to produce several molecules, such as SCFAs and peptidoglycan, which affect the innate immune system [176]. Prebiotics may enhance insulin resistance and glucose tolerance [177] and reduce intestinal inflammation, endotoxemia, and cytokines which might be beneficial in sepsis. As such, desaminotyrosine (DAT) maintains mucosal immunological homeostasis and barrier integrity, and reduces mucosal inflammation in DSS-induced endotoxemia and septic shock in rodents [178]. Some prebiotics from the Chinese herbs, Xuanbai Chengqi decoction (XBCQ), also attenuates pulmonary infection in rodents through the improved gut barrier function and promoted survival [179,180]. In addition, Finger Millet arabinoxylan (FM-AX), a non-starch polysaccharide produced from cereals, demonstrates attenuates endotoxemia in mice via reduction of high-fat diet-induced leaky gut [181]. In human studies, prebiotics reduces the incidence of sepsis, mortality, and length of hospital stay in premature infants [182]. While the preparation cost for FMT and probiotics is usually high with sophisticated technology due to the management of the viable organisms, prebiotics preparation seems to be less expensive with, perhaps longer shelf life. However, prebiotics cannot promote the growth of bacteria that do not present in the gut, and most commercially available products are a combination of prebiotics with probiotics. Due to the less expensive process of preparation, the prebiotics, a single or in combination, selectively promotes the growth of beneficial bacteria that commonly found in the host in sepsis is interesting. More studies are warranted.

## Conclusions

Gut leakage and changes in the intestinal microbiome in sepsis are the consequence of intestinal immunity defects caused by intestinal hypoperfusion, immune cell apoptosis, and enteric neuro-humoral-immunity responses. The increased abundance of pathogens in the bacterial microbiome associated with a leaky gut may result in the translocation of microbial molecules and even viable microorganisms, eventually worsening the course of sepsis. Despite several previous reviews on gut microbiota in sepsis [183–186], the collection of data on gut mycobiome (fungiome) and virobiome is usually limited to the non-sepsis condition [187–190] and the review of gut microbiome together with leaky gut in sepsis is still less. Here, the close correlation between gut microbiota (bacteria, fungi and viruses) and sepsis severity also suggests that attenuation of leaky gut and gut dysbiosis might be a target of future adjunctive therapies. Moreover, the role of virome, mycobiome, as well as novel metagenomics of microbial identification must be in the pipeline of the future research areas and are urgently needed fields.

## Data Availability

Data sharing is not applicable to the review.

## Abbreviations

DAMP: damage-associated molecular pattern
DAT: desaminotyrosine
ESBL: extended-spectrum β-lactamase
FM-AX: Finger Millet arabinoxylan
FMT: faecal microbiota transplantation
GBS: gut–brain–skin
NSAIDs: non-steroidal anti-inflammatory drugs
XBCQ: Xuanbai Chengqi decoction
